# Longitudinal development of the airway microbiota in infants with cystic fibrosis

**DOI:** 10.1038/s41598-019-41597-0

**Published:** 2019-03-26

**Authors:** Bushra Ahmed, Michael J. Cox, Leah Cuthbertson, Phillip James, William O. C. Cookson, Jane C. Davies, Miriam F. Moffatt, Andrew Bush

**Affiliations:** 1 National Heart and Lung Institute, Imperial College London, London, UK; 2 Department of Respiratory Paediatrics, Royal Brompton Hospital, London, UK

## Abstract

The pathogenesis of airway infection in cystic fibrosis (CF) is poorly understood. We performed a longitudinal study coupling clinical information with frequent sampling of the microbiota to identify changes in the airway microbiota in infancy that could underpin deterioration and potentially be targeted therapeutically. Thirty infants with CF diagnosed on newborn screening (NBS) were followed for up to two years. Two hundred and forty one throat swabs were collected as a surrogate for lower airway microbiota (median 35 days between study visits) in the largest longitudinal study of the CF oropharyngeal microbiota. Quantitative PCR and Illumina sequencing of the 16S rRNA bacterial gene were performed. Data analyses were conducted in QIIME and Phyloseq in R. *Streptococcus* spp. and *Haemophilus* spp. were the most common genera (55% and 12.5% of reads respectively) and were inversely related. Only beta (between sample) diversity changed with age (Bray Curtis r^2^ = 0.15, *P* = 0.03). *Staphylococcus* and *Pseudomonas* were rarely detected. These results suggest that *Streptococcus* spp. and *Haemophilus* spp., may play an important role in early CF. Whether they are protective against infection with more typical CF micro-organisms, or pathogenic and thus meriting treatment needs to be determined.

## Introduction

Respiratory infections begin early in CF even in the absence of symptoms^1^. More frequent exacerbations before 2 years of age are associated with reduced forced expiratory volume in one second (FEV_1_) at 5 years^2^. The pathophysiology of respiratory infections, however, remains poorly understood. Based on conventional culture techniques, organisms such as *Staphylococcus aureus* and *Pseudomonas aeruginosa* dominate. In the UK anti-staphylococcal prophylaxis is recommended for all CF infants. 16S rRNA gene sequencing, however, has revealed that the airways of patients with CF harbour rich communities of microbiota including anaerobes such as *Veillonella* spp.^3,4^ and the *Streptococcus anginosus* group^5^, which may be harmful, or protective by preventing the growth of more virulent organisms. Complex interactions between microorganisms are likely to be important^5–7^.

Adult studies have demonstrated an inverse relationship between microbial diversity and disease progression^8,9^. Whether reduced diversity is causative of end-stage disease or an effect of frequent antibiotics has yet to be determined. Studies in very early childhood may help identify strategies for maintaining lung health into adulthood.

Longitudinal studies of the airway microbiota in CF infants are challenging. Infants cannot spontaneously expectorate sputum and repeated sampling of the lower airway is neither feasible nor ethical. Longitudinal studies of bronchoalveolar lavage fluid (BAL) from CF infants thus far have taken samples every 6 months^10^ or annually^11^, thus limiting their ability to relate microbiota changes to symptoms. Nasopharyngeal samples, used to track changes in the airway microbiota in the first year of life, have demonstrated differences between CF infants and controls, with *Staphylococcus spp*. and *Streptococcus spp*. more prevalent in CF and *Haemophilus spp*. and *Prevotella spp*. more prevalent in controls^12^. Studies comparing nasopharyngeal sampling with sputum, however, have shown significant differences in both community diversity and composition between these two respiratory samples in children with CF^13^. Although an imperfect surrogate, oropharyngeal samples have shown better correlation with the lower airway microbiota^14,15^.

Larger data sets, coupling detailed clinical information with frequent sampling, are needed to better understand the progression of the CF airway microbiota. This present study aimed to describe how the CF infant microbiota develops over the first two years of life.

## Results

### Patient demographics

Thirty infants with CF were recruited (Table 1) and followed-up for a mean duration of 14 months (SD 5 months) with a median of 35 days (range 1–301 days) between each sample visit. Two patients (7%) were lost to follow up and three patients had only 6 monthly or annual follow up at the Royal Brompton Hospital (RBH). For individual sampling frequency see Supplementary Fig. S1. Due to being opportunistic, sampling frequency varied (Supplementary Fig. S1a) and consequently samples were clustered into age ranges (e.g. 3–4 months etc.) (Supplementary Fig. S1b). Where a child had more than one sample in a given time period, the first sample taken in that range was used.

**Table 1:** Patient demographics.

Demographic (N = 30)	Median (range) or N (%)
**Age at recruitment (in days)**	84 (35–235)
**Gender (female)**	17 (57%)
**CFTR Genotype**	
Homozygous p.Phe508Del	12 (40%)
Heterozygous p.Phe508Del	17 (57%)
Other	1 (3%)
**Extra-pulmonary features**	
Pancreatic insufficiency	22 (73%)
Meconium ileus	1 (3%)
GORD	8 (27%)
**Birth history**	
**Mode of delivery**	
Vaginal delivery	21 (70%)
Caesarian section	6 (20%)
**Feeds in infancy**	
Breastfeeding (exclusively or in combination with formula)	16 (53%)
Exclusively formula fed	14 (47%)
**Exacerbations & antibiotic use**	
**Exacerbations**	7 (23%)
**Prophylactic antibiotics at recruitment**	25 (83%)
**Bacterial growth on TS culture**	
*P. aeruginosa*	7 (23%)
*S. aureus*	1 (3%)
*H. influenzae*	1 (3%)
Upper respiratory tract flora	1 (3%)

CFTR – cystic fibrosis transmembrane conductance regulator; GORD - gastro-oesophageal reflux disease; TS - throat swab. *P. aeruginosa* – *Pseudomonas aeruginosa. S. aureus – Staphylococcus aureus. H. influenzae – Haemophilus influenzae*.

Flucloxacillin was the first line prophylaxis in all participants. Seven patients did not tolerate Flucloxacillin and were later changed to Amoxicillin and Clavulanic acid (Co-amoxiclav) prophylaxis. Seven patients (23%) had one pulmonary exacerbation each. *P. aeruginosa* was the most commonly grown organism (23%, all “scanty” growths [=10^3^ CFU/ml]) leading to treatment with three weeks of oral Ciprofloxacin and one month of Tobramycin nebulisers. Three patients who grew *P. aeruginosa* also received intravenous (IV) antibiotics.

### Examining sequencing quality

Two hundred and forty one throat swabs (TS) were sequenced from thirty patients (median of nine samples/patient [range 1–15 samples]). 14,529,156 high quality reads were obtained.

Operational Taxonomic Units (OTUs) were identified as contaminants if they were abundant in the controls or had been identified as common reagent contaminants in previous literature^16^. The following organisms were consequently removed: *Undibacterium* spp., *Comamonadaceae*, *Sediminibacterium* spp., *Methylobacterium* spp., *Planomicrobium* spp. and a *Burkholderia* OTU (ID 1931).

### Development of the airway microbiota in the first 2 years of life

A median of 48,877 copies of the 16S rRNA gene were obtained per swab (range 20–22,461,921 copies/swab). Bacterial load increased significantly between 12–21 months of age (Parameter estimate = 1.7, z-value = 2.3, standard error = 0.8, *P* = 0.022).

**Figure 1:**
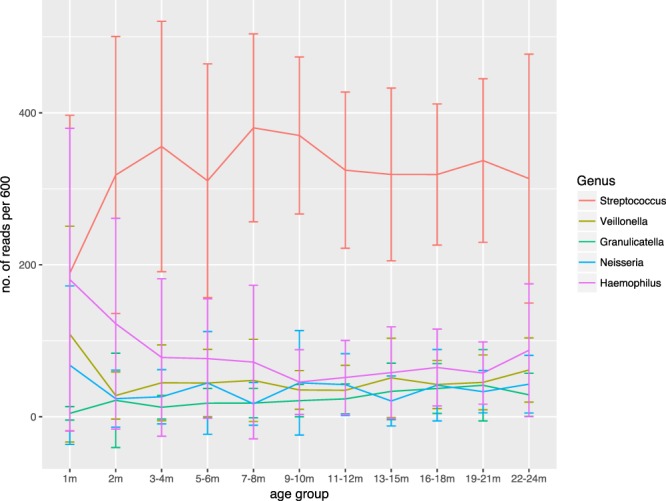
Changes in the relative abundance of the five most common genera with age in infants with CF. Figure shows the mean relative abundance of *Streptococcus* spp., *Haemophilus* spp., *Neisseria* spp., *Veillonella* spp. and *Granulicatella* spp. using a proportional scale where all samples have been rarefied to 600 reads. The error bars represent the standard deviation. An inverse relationship in the relative abundance of *Streptococcus* spp. and *Haemophilus* spp. in the first two years of life is observed.

*Streptococcus* was the most common genus, representing over half the bacterial community (55% of reads). Other common genera included: *Haemophilus* (12.5%), *Veillonella* (7.4%) and *Neisseria* (5.6%). *Staphylococcus* spp. and *Pseudomonas* spp. had low relative abundance (0.1%). Examining trends in relative abundance of the five most common genera from baseline, *Streptococcus* spp. and *Haemophilus* spp. began with a similar relative abundance but diverged thereafter (Fig. 1). *Streptococcus* spp. increased in the first 7–8 months of life before reaching an asymptote (*P* < 0.001) (Table 2). In contrast, *Haemophilus* spp. decreased from 3 to 9 months of age and thereafter plateaued (*P* = 0.034). *Granulicatella* spp. gradually increased over the first two years of life (*P* < 0.001 at 19–21 months of age). *Veillonella* spp. decreased rapidly at 2 months of age (*P* < 0.001) and then remained at a similar relative abundance whereas the relative abundance of *Neisseria* spp. fluctuated.

**Table 2:** Summary of genus level changes for 5 most common organisms with age.

Organism	Age range (in months)	Estimate	z-value	SE	*P*-value
*Streptococcus*	7–8	0.77	3.6	0.22	<0.001
*Haemophilus*	9–10	−1.1	−2.1	0.50	0.034
*Veillonella*	2	−1.5	−3.5	0.44	<0.001
*Neisseria*	7–8	−1.6	−2.9	0.57	0.004
*Granulicatella*	19–21	2.3	3.7	0.62	<0.001

Shown above is the age range for each of the most common organisms at which the greatest change in relative abundance was seen using a non-linear mixed effects model using a negative binomial error structure controlling for patient. The model fits changes in abundance using a log scale. The mean change in relative abundance from baseline is shown by the “Estimate”. The z-value indicates the number of standard deviations of the estimate from the mean relative abundance at baseline. SE – standard error.

**Figure 2:**
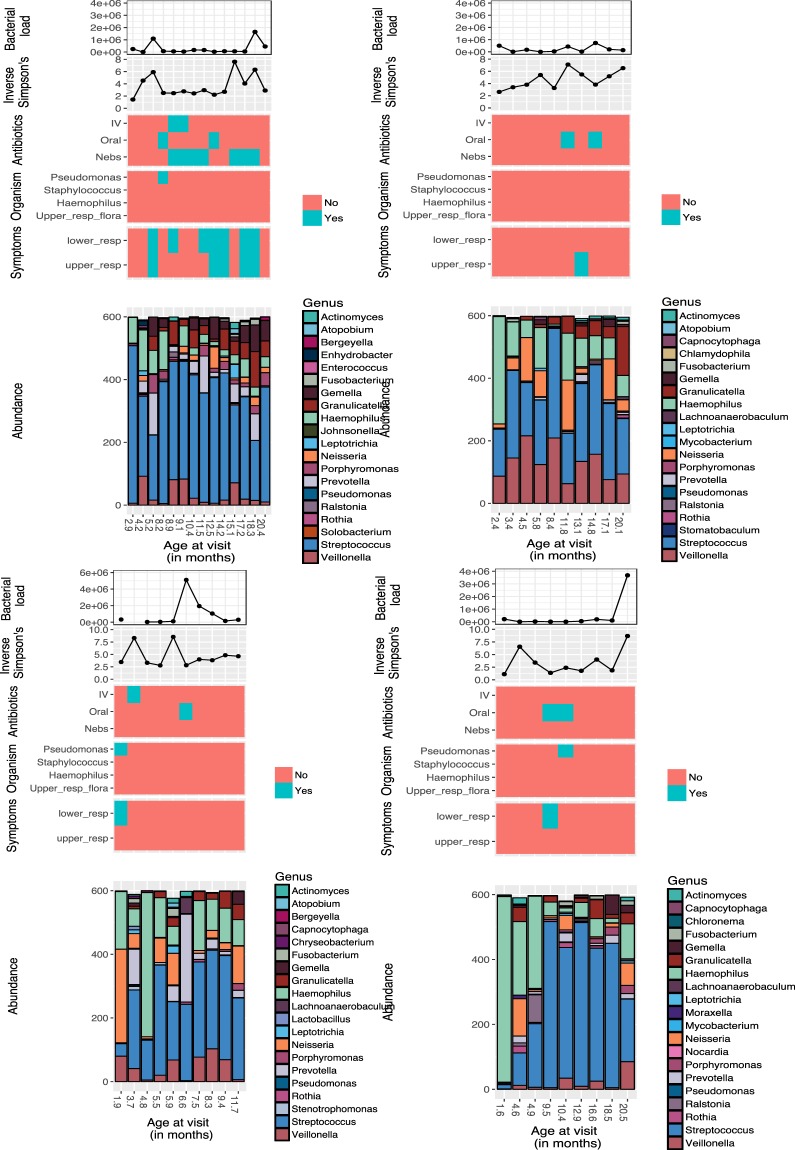
Example individual patient barplots illustrating changes with age (in months) in relative abundance of genera, bacterial load (16S rRNA copies per swab), alpha diversity changes (measured by Inverse Simpson’s) and clinical variables. Clinical variables illustrated include: antibiotic administration at the time of sample collection (Intraveous [IV], oral and nebulised [nebs]); bacterial culture results, and presence of respiratory tract symptoms at the time of sample collection. All infants were on prophylactic antibiotics at the time of sample collection. Four individual patient barplots are shown. Little change was seen in bacterial load, the Inverse Simpson’s diversity index or community structure (shown by the barplot) with changes in symptoms, growth of *P.aeruginosa* or antibiotic treatment.

Several OTUs showed a small (r < 0.3) but significant (*P*_adj_ < 0.05) increase in relative abundance with age, including several *Streptococcus* (IDs 412, 432 and 1841) and *Haemophilus* (IDs 2347 and 2369) OTUs (Table 3). One *Streptococcus* OTU (ID 1897) showed a decrease in relative abundance. Examining individual patient barplots revealed the airway microbiota was highly individual. No consistent changes were seen with changes in symptoms or positive growth on bacterial cultures (Fig. 2).

**Table 3:** List of OTUs showing significant (*P*_adj_ < 0.05) changes in relative abundance with age.

OTU name	r value	Adjusted *P-*value
*Actinomyces* (OTU ID 2665)	0.25	<0.001
*Alysiella* (OTU ID 2166)	0.23	0.006
*Atopobium* (OTU ID 1256)	0.21	0.013
*Capnocytophaga* (OTU ID 812)	0.20	0.016
*Enterococcus* (OTU ID 1712)	0.20	0.014
*Haemophilus* (OTU ID 2347)	0.23	0.006
*Haemophilus* (OTU ID 2369)	0.24	0.003
*Lachnoanaerobaculum* (OTU ID 251)	0.26	<0.001
*Lactobacillus* (OTU ID 1586)	0.19	0.025
*Lautropia* (OTU ID 2253)	0.26	<0.001
*Leptotrichia* (OTU ID 1059)	0.26	<0.001
*Neisseria* (OTU ID 2124)	0.19	0.025
*Prevotella* (OTU ID 657)	0.26	<0.001
*Prevotella* (OTU ID 692)	0.19	0.020
*Rothia* (OTU ID 2603)	0.21	0.013
*Streptococcus* (OTU ID 412)	0.23	0.006
*Streptococcus* (OTU ID 432)	0.25	0.002
*Streptococcus* (OTU ID 1841)	0.24	0.004
*Streptococcus* (OTU ID 1897)	−0.19	0.016
*Veillonella* (OTU ID 1463)	0.24	0.006

Assessed using Spearman rank multiple correlation testing with FDR correction. Several species of *Streptococcus* spp., *Haemophilus* spp. and *Prevotella* spp., as well as other individual species showed a small (r < 0.3) but significant positive correlation with age in the first two years of life.

**Figure 3:**
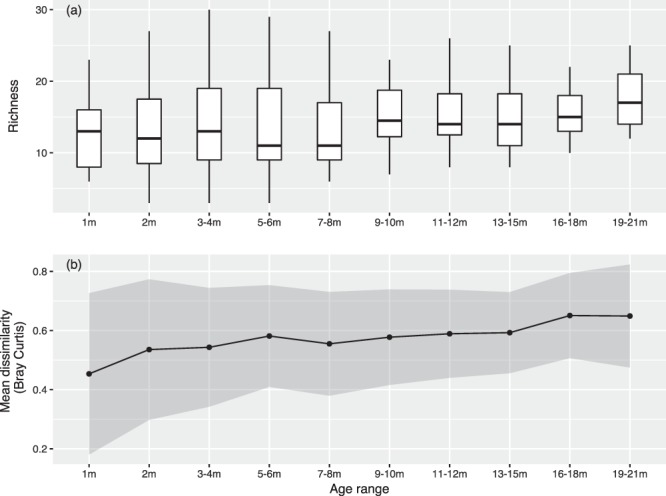
Changes in diversity with age. (**a**) Boxplot illustrating changes in alpha diversity measured by species richness for each age group. Using a non-linear mixed effects model with a negative binomial distribution, there was no significant association between richness and age (*P* > 0.05). (**b**) Change in beta diversity with age measured using the Bray Curtis dissimilarity score. Mean and standard deviation in Bray Curtis dissimilarity score shown for each age group. This demonstrates an increase in dissimilarity by age (PERMANOVA, r^2^ = 0.15, *P* = 0.03).

There was no significant change in alpha diversity with increasing age (species richness, *P* > 0.05) (Fig. 3a). A significant influence of patient age on community structure was observed (beta diversity, Bray Curtis dissimilarity, r^2^ = 0.15, *P* = 0.03) with samples becoming less similar with age (Fig. 3b).

Alpha and beta diversity testing were repeated to determine whether any clinical variables (full listing in Supplementary Material) influence community structure. Evenness and the Shannon diversity index were significantly higher in patients delivered by Caesarian section than vaginal delivery, although differences were small (e.g. Shannon diversity index median 0.4 [0.1–0.6] for Caesarian section and 0.345 [0.02–0.63] for vaginal delivery, *P* = 0.045]). Similarly, whilst several clinical variables had a significant influence on beta diversity, the degree of variance attributable was small (<3%) (Table 4). In contrast, 18% of the variation in beta diversity was explained by the patient the sample was from (Bray Curtis, r^2^ = 0.18, *P* = 0.001).

**Table 4:** Influence of clinical variables on beta diversity.

Clinical variable	r^2^	*P*-value
Mode of delivery	0.02	0.009*
Type of feeds (breast, bottle or mixed)	0.006	0.264
Homozygous p.Phe508Del	0.02	0.004*
Pancreatic insufficiency	0.009	0.06
Gastro-oesophageal reflux disease	0.005	0.215
Upper respiratory tract symptoms (yes/no)	0.01	0.027*
Lower respiratory tract symptoms (yes/no)	0.004	0.327
Any *P. aeruginosa* grown	0.01	0.015*
Nebulised antibiotics	0.01	0.039*
Oral antibiotics	0.01	0.006*

*Denotes clinical variables which exerted a significant influence (*P* < 0.05) on the Bray Curtis dissimilarity score as measured by permutational multivariate ANOVA (PERMANOVA).

### Changes in the microbiota with the first exacerbation or growth of *P. aeruginosa*

Seven patients experienced a pulmonary exacerbation during the study period. Five patients had sequential samples collected prior to, during and following treatment of an exacerbation. Consequently only an exploratory analysis was performed. Samples were compared at:

- Baseline (B) – within 1 month prior to admission for IV antibiotics
- Exacerbation (E) – within 48 hours of starting IV antibiotics
- Treatment (T) – at 10–14 days of IV antibiotics
- Recovery (R) – within 1 month after completing 2 weeks of IV antibiotics

Overall, there was no significant differences in bacterial load or community composition with the first exacerbation at B, E, T and R timepoints (Supplementary Material, Supplementary Fig. S2).

Seven patients grew *P. aeruginosa* during the study period. Six had sequential samples prior to, during and after *P. aeruginosa* growth following treatment. Exploratory analysis of these patients revealed a low relative abundance (<1%) of *Pseudomonas* spp. at time of *P. aeruginosa* growth on culture. There was a significant difference in beta diversity only with growth of *P. aeruginosa* (Bray Curtis dissimilarity: r^2^ = 0.135, *P* = 0.036) (Supplementary Material, Supplementary Fig. S3).

## Discussion

In this study we have shown that the airway microbiota in CF infants is highly individual and develops gradually over the first two years of life. No relationship between age and alpha (within sample) diversity of the airway microbiota was found. A pronounced effect of age was however seen on beta (between sample) diversity, a measure of community nestedness and turnover, with 15% of variance attributed to the age of the patient with an increase in dissimilarity seen with age. This suggests that the oropharyngeal microbiota is less homogenous with age in infants with CF. Similarly, bacterial load increased between 12 and 21 months of age. This suggests there may be an accrual of organisms in the first two years of infancy although the prognostic significance of this will only be known through follow-up.

*Streptococcus* spp. and *Haemophilus* spp. were the most common genera present in the first two years of life (55.0% and 12.5% of total reads respectively). These high detection rates by sequencing were not reflected in the bacterial culture results and neither genera are thought to be particularly important clinically in CF. This confirms that bacterial cultures may fail to identify organisms present in the airway microbiota, even when they are highly abundant^17^. This has previously been demonstrated for the *Streptococcus anginosus* group at exacerbation^18^. Streptococcal species are often dismissed as “upper respiratory tract flora”, even when detected, but only one participant grew “upper respiratory tract flora” during this present study, so this is unlikely to be the explanation for the findings.

Over the first 2 years of life, trends in the relative abundance of *Streptococcus* spp. and *Haemophilus* spp. appear to have an inverse relationship: as the relative abundance of *Streptococcus* spp. increased in the first 9 months of life, the relative abundance of *Haemophilus* spp. decreased. Thus *Streptococcus* spp. and *Haemophilus* spp. may have an antagonistic relationship in the airways of CF infants. It is interesting to note that studies of the nasopharyngeal microbiota in CF infants have illustrated a higher relative abundance of *Streptococcus* spp. in CF and *Haemophilus* spp. in controls^19^. *In vitro* and when co-cultured, during its stationary phase of growth planktonic *Streptococcus pneumoniae* outcompetes *Haemophilus influenzae*, particularly, in low pH environments^20^. A pig model of CF lung disease has demonstrated that airway surface liquid (ASL) is more acidic in CF due to defective bicarbonate transport than in those with normal CFTR function resulting in reduced bacterial killing^21^. It is possible therefore, that in the airways of CF infants, low ASL pH creates an environment favouring overgrowth of *Streptococcus* spp. over *Haemophilus* spp. Communities with a higher relative abundance of *Streptococcus* spp. have been associated with less airway inflammation in CF measured by lower total cell counts and fewer neutrophils in BAL^22^. Cellular analysis could not be performed in this study to explore the relationship between the microbiota and airway inflammation as all our samples were immediately frozen on dry ice to prevent changes in the microbiota^23^, thus precluding cellular and cytokine analysis. Further data are required to explore this as well as determining whether the increase in *Streptococcus* species may pave the way for infection with more virulent organisms.

More commonly identified on bacterial cultures in children with CF, *Staphylococcus* spp. and *Pseudomonas* spp. had very low relative abundance in the microbiota in the first two years of life. However, it should be noted that all the children studied received prophylactic anti-staphylococcal antibiotics throughout the study period in accordance with UK CF Trust Guidelines^24^. It is possible that the low levels of *Staphylococcus* spp. seen reflect the suppression of *Staphylococcus* spp. growth due to this prophylaxis, the effects of which on the airway microbiota are uncertain.

Similarly, the influence of anti-staphylococcal prophylaxis on the relative abundance of *Streptococcus* spp. is unclear. Suppression of *Staphylococcus* spp. growth through use of flucloxacillin may create an environment which enables *Streptococcus* spp. to thrive. A higher relative abundance of *Staphylococcus* spp. was demonstrated in one cross-sectional study from Australia comparing 20-year old historical BAL samples in thirteen infants with CF not on antibiotic prophylaxis with nine control infants being investigated for stridor^25^. However, examining the composition of the microbiota of individual patients in this Australian cohort, *Streptococcus* spp. was also one of the most common organisms present in the majority of stable infants with CF, often in greater abundance than *Staphylococcus* spp. Furthermore, a comparison of BAL from 32 infants with CF on prophylaxis with those not on prophylaxis from Australia and USA respectively did not demonstrate an association between prophylaxis use and the relative abundance of specific genera, with the exception of *Fusobacterium* spp., which was increased in those not on prophylaxis^26^. A large cross-sectional comparison of BAL from 146 children and adults with CF from USA who were not on prophylaxis demonstrated that *Streptococcus* spp. was the most abundant organism in those under 2 years of age and decreased with age, similar to the findings in this study^22^. Thus, *Streptococcus* spp. appears to be a dominant organism in the airways of infants with CF regardless of prophylaxis use or sampling method (BAL or TS) and its role in CF lung disease warrants further exploration.

*Pseudomonas* spp. was an uncommon genus present in the samples taken from patients at the time of *P. aeruginosa* growth (relative abundance of 0.1%). It is highly unlikely that this represents an error in sequencing as *Pseudomonas* spp. was readily sequenced from the mock communities included in each sequencing run. This, however, most likely reflects the high sensitivity of bacterial cultures for isolating *P. aeruginosa*, even when it is present in small numbers and the success of clinical microbiology protocols in actively seeking to identify *P. aeruginosa* when present. It may also reflect the ability of *P. aeruginosa* to outgrow other organisms when co-cultured, highlighting the potential lack of bias when performing 16S rRNA gene sequencing to analyse the airway microbiota. Additionally, in this study overgrowth of *P. aeruginosa* on samples for culture independent analysis was limited by rapid freezing of samples at the point of collection.

Pulmonary exacerbations are recognised to be an important determinant of later FEV_1_ in children with CF^2^. Whilst no overall changes in the microbiota were seen with exacerbations, this is likely due to the small numbers of patients who met the definition of pulmonary exacerbation used in this study. This limited the analyses to those patients with the most severe exacerbation who required IV antibiotics and may have missed changes in patients with milder symptoms who were treated with oral antibiotics but may still have had significant lung function decline^27^. Use of oral antibiotics was not included for two reasons. Firstly, because in current clinical practice there is a low threshold for initiating oral antibiotic treatment for even mild respiratory symptoms. Secondly, because this relied on retrospective parental reports of oral antibiotic usage in which there was a lack of confidence. Individual patient barplots, tracking changes in the microbiota with clinical variables, did not identify a bacterial biomarker associated with changes in symptoms necessitating oral or IV antibiotics. Larger sample sizes are needed to explore further changes occurring at pulmonary exacerbation.

An exploratory analysis, as sample size was small, was conducted to investigate whether changes in the microbiota occur prior to first *P. aeruginosa* growth, at the time of growth and following treatment for *P. aeruginosa*. Samples at each of these timepoints were compared within patients. Whilst there was no significant difference in bacterial load or alpha diversity, between samples difference in beta diversity was seen. This suggests that there are differences in community structure with growth and treatment of *P. aeruginosa* but larger studies are needed to explore these findings further.

To our knowledge, this is the largest prospective, longitudinal study of the oropharyngeal microbiota in infants with CF to date with more frequent sampling (median 5 weeks between samples) than previously reported and consequently has resulted in greater opportunity to identify changes in the microbiota with symptoms. At each timepoint, patient sampling was paired with collection of detailed clinical data resulting in several variables, such as mode of delivery, being identified as important factors exerting a small but nonetheless significant influence on the airway microbiota in the first 2 years of life.

There are, however, several important limitations in this study. Firstly, whilst the number of participants studied is larger than previously reported, this study is still relatively small. During the 18-month recruitment period, the families of thirty infants out of thirty-five (86%) diagnosed with CF consented to take part in the research thus obtaining a greater sample size was not realistically feasible. Nevertheless despite the small sample size, changes in the microbiota with age were seen and the additional findings highlight that a further multi-centre study is warranted.

Another limitation of this study is that it was not feasible to include a healthy control group due to practical difficulties in recruiting healthy babies and ensuring regular, frequent attendances for TS collection. It is difficult to know therefore whether changes seen in this study are related to CF disease processes or may be normal changes occurring in infants during the first 2 years of life of infants. *Streptococcus* and *Haemophilus* have been found to be common organisms in one study of the nasopharyngeal microbiota in the first two years of life in 60 healthy infants, with notably *Haemophilus* replacing earlier *Staphylococcus*-dominated profiles with age^28^. Whether the healthy oropharyngeal microbiota shows similar changes is unknown.

A common limitation of longitudinal study of the airway microbiota in infants is the difficulty in obtaining frequent lower airway samples. Infants cannot spontaneously expectorate sputum and BAL cannot be performed frequently due to the need for a general anaesthetic. For this reason, TS were used in this study as previous work from our group demonstrated that TS are an imperfect but reasonable surrogate for lower airway sampling (BAL or bronchial brushings) in children with chronic lung diseases^15^. This included twelve infants with CF from this cohort who had a bronchoscopy between three and five months of age. Nonetheless, microbiota profiles in TS are not identical to lower airway samples. Comparing upper airway samples and BAL from seventeen infants with CF, one study demonstrated comparable relative abundance of *Streptococcus* spp. but reduced *Staphylococcus* spp. between oropharyngeal samples and BAL^19^. Thus, it is possible that the low relative abundance of *Staphylococcus* spp. seen in this study may be influenced by the sampling method.

Ideally the participants in this study would have been recruited as soon as possible following diagnosis i.e. within the first 1–2 months of life. Whilst many families are admitted for 2 days following diagnosis for education, it was felt to be insensitive to attempt to recruit participants during this highly emotional time. Consequently, participants were therefore recruited at their next routine clinical appointment following their educational visit. This resulted in variability in the age of recruitment. In addition, patient sampling was performed opportunistically during routine clinical appointments by a single operator. Whilst this ensured consistency in sampling, it did sometimes result in missed samples at a given timepoint.

In addition, all of the CF children in this study received anti-staphylococcal prophylaxis which may have impacted community structure. This prophylaxis is not a policy adopted worldwide and the benefit of antibiotic prophylaxis in children with CF is still under debate. Therefore, a future study comparing longitudinal changes in the microbiota between infants with CF on antibiotic prophylaxis with those receiving no prophylaxis, such as in the CF START study (www.cfstart.org.uk), would provide real insights into the role of antibiotic prophylaxis in airway infections in CF.

Despite this, the results of this present study suggest that *Streptococcus* spp. and *Haemophilus* spp. may play an important role in early CF. Whether either or both are protective against infection with more typical CF micro-organisms, or pathogenic and thus meriting treatment needs to be determined.

## Methods

For additional details see Supplementary Material.

### Study subjects

Patients with a diagnosis of CF confirmed on NBS were recruited opportunistically at RBH from December 2012–March 2014. All participants were prescribed prophylactic anti-staphylococcal antibiotics during the study period. The study was approved by the RBH Biomedical Research Unit Advanced Lung Disease Biobank (NRES reference 10/H0504/9) and all methods performed in accordance with the relevant guidelines and regulations. Informed written consent and age-appropriate assent was obtained from parents and children respectively.

### Study design

TS and paired clinical information (see Supplementary Material) were prospectively collected during routine clinical appointments for up to two years. An exacerbation was defined as a change in symptoms necessitating an admission to hospital for intravenous (IV) antibiotics (determined by paediatricians blinded to the microbiota results).

Using cotton-tipped sterile swabs TS were collected by a single operator. Technical controls were taken to test for contamination. All samples were immediately frozen on dry ice and stored at −80 °C until DNA extraction. Microbial culture was performed as per standard clinical practice for CF samples in accordance with CF Trust Guidelines^29^.

### 16S rRNA gene library preparation and sequencing

DNA was extracted from frozen swab heads using the MP Bio FastDNA Spin Kit for Soil (http://www.mpbio.com). Blank controls with no sample added were taken from each DNA extraction kit to test for contamination^16^.

PCR of the 16S rRNA V4 region was performed in quadruplicate using a custom indexed forward primer S-D-Bact-0564-a-S-15 (5′ AYT GGG YDT AAA GNG 3′), reverse primer S-D-Bact-0785-b-A-18 (5′ TAC NVG GGT ATC TAA TCC 3′) and a high fidelity *Taq* polymerase master mix (Q5, New England Biolabs). Primer sequences were based on Klindworth *et al*.^30^, with dual-barcoding as per Kozich *et al*.^31^ with adaptors from Illumina.

A mock community (Table S1) was included to assess sequencing quality. PCR cycling conditions were: 95 °C for 2 minutes followed by 35 cycles of 95 °C for 20 seconds, 50 °C for 20 seconds and 72 °C for 5 minutes. Amplicons were purified, quantified and equi-molar pooled and the library paired-end sequenced (Illumina MiSeq V2 reagent kit)^31^ as previously described^15^.

### 16S rRNA gene quantitative PCR (qPCR)

Bacterial load was quantified using KAPA BioSystems SYBR Fast qPCR Kit with the same 16S rRNA V4 primers used for sequencing. See Supplementary Material for further details of qPCR conditions.

### Analysis

Sequence processing was performed in QIIME (version 1.9.0)^32^. Community level differences in alpha and beta diversity and OTU level differences were analysed using Phyloseq in R (version 3.2.0).

The sequencing depth differed widely between samples. To maintain power as many sequential samples as possible were retained by using two different approaches: for alpha diversity using mixed effects models, random re-sampling with replacement whilst for beta diversity using permutational multivariate ANOVA using the adonis function in Phyloseq, samples were rarefied to 600 reads. See Supplementary Material for further details of the rationale for this approach.

Non-linear mixed effects modelling using a negative binomial distribution was performed using the glmmADMB package in R^33^ controlling for patient to assess the relationship between age (clustered into age-ranges) and: (a) bacterial load; (b) alpha diversity, and (c) the relative abundance of the most common genera agglomerated by genus (See Supplementary Material).

Between sample beta-diversity differences (measured using Bray Curtis dissimilarity) were tested using a permutational multivariate ANOVA (adonis)^34^ blocked by participant study number. OTU level changes were assessed using multiple correlation testing using Spearman’s rank with a false discovery rate (FDR) correction. A *P* value of less than 0.05 was considered statistically significant. Sequence data is available at the ENA (Accession number: PRJEB26618).

## Supplementary information

Supplementary material: Longitudinal development of the airway microbiota in infants with cystic fibrosis

## Data Availability

Sequence data is available at the ENA (Accession Number: PRJEB26618).
