# Supplementary material for: Longitudinal development of the airway microbiota in infants with cystic fibrosis
Source: Sci Rep. 2019 Mar 26;9:5143. doi: 10.1038/s41598-019-41597-0 (PMC6435666; doi:10.1038/s41598-019-41597-0)
Supplement: Supplementary file 1 — Supplementary material: Longitudinal development of the airway microbiota in infants with cystic fibrosis [file 41598_2019_41597_MOESM1_ESM.doc]

**Supplementary material:**

**Longitudinal development of the airway microbiota in infants with cystic fibrosis**

Bushra Ahmed^1, 2*^, Michael J. Cox^1^, Leah Cuthbertson^1^, Phillip James^1^, William O.C. Cookson^1^, Jane C. Davies^1, 2^, Miriam F. Moffatt^1†^ and Andrew Bush^1, 2†^.

^1^National Heart and Lung Institute, Imperial College London, London, UK; ^2^Department of Respiratory Paediatrics, Royal Brompton Hospital, London, UK.

**Methods**

**Throat swab sampling**

Throat swabs were collected using a sterile, cotton-tipped swab (Sterilin, UK) by rubbing a swab back and forth 5 times in front of the uvula, whilst taking care not to touch any other oropharyngeal surface [^1^](#_ENREF_1),[^2^](#_ENREF_2). A tongue depressor was used to limit contamination from the tongue and oral mucosa. All samples were placed immediately onto dry ice and transferred to -80^o^C where they were stored until DNA extraction.

**Clinical information**

The following clinical information was collected at baseline only: age; gender; CFTR genotype; mode of delivery (vaginal delivery or Caesarian section); gestation at birth, and meconium ileus at birth.

At each study visit the following clinical information was collected: length (in cm), weight (in kg); presence of complications and co-morbidities, including pancreatic insufficiency and gastro-oesophageal reflux disease (diagnosed either clinically or on positive pH study); presence of upper or lower respiratory symptoms at the time of sample collection (yes/no); current antibiotic use (oral prophylaxis, oral treatment courses, nebulised and intravenous antibiotics, yes/no) and duration; feeding history (formula or breastfed, including age at weaning); medication history, and paired clinical bacterial culture results.

**16S rRNA gene quantitative PCR (qPCR)**

Bacterial load was quantified using the SYBR Fast qPCR Kit (KAPA BioSystems). A standard curve was constructed using a full length 16S rRNA gene of *Vibrio natriegens* DSMZ 759 cloned into TOPO TA vector at with 5 dilutions ranging from a concentration of 10^4^ to 10^8^molecules/μl. Sample DNA was diluted 1:5 in PCR grade water to a total volume of 25 μl per sample. Custom 16S rRNA gene primers were used (Illumina)[^3^](#_ENREF_3) targeting the V4 hypervariable region : forward primer S-D-Bact-0564-a-S-15 : 5’ AYT GGG YDT AAA GNG 3’, and reverse primer S-D-Bact-0785-b-A-18 : 5’ TAC NVG GGT ATC TAA TCC 3’. The same primers and target region were subsequently used for sequencing.

Sample reactions were set up in MicroAmp Fast-96 well reaction plates placed in a splash free support base to protect the SYBR green reagent from the light. Bacterial DNA within samples was quantified in 15 μl triplicate reactions as follows:

- 7.5 μl of SYBR Fast qPCR Master Mix
- 0.3 μl of each F and R primer (10 μM)
- 1.9 μl PCR grade water
- 5 μl of template (bacterial standard, non-template control [PCR grade water] or sample DNA)

Plates were sealed with a MicroAMP Optical Adhesive Seal and qPCR reactions were run on the ViiA7 Real-Time PCR system (Applied Biosystems/Thermo Fisher Scientific, Waltham, USA) with the following conditions:

- 90^o^C for 3 minutes

followed by 40 cycles of:

- 95^o^C for 20 seconds
- 50^o^C for 30 seconds
- 72^o^C for 30 seconds

Quantification was deemed reliable if a reaction efficiency of 60-70%, R^2^ > 0.998 was attained and if the library dilutions amplified within the dynamic range of the assay. Bacterial DNA was quantified from the standard curve.

**Table S1: Mock community composition**

| **DSM Number** | **Species** |
| --- | --- |
| 7288 | *Burkholderia cepacia* |
| 19748 | *Chlamydophila pneumoniae* |
| 20478 | *Enterococcus faecalis* |
| 30083 | *Escherichia coli* |
| 15643 | *Fusobacterium nucleatum subsp. nucleatum* |
| 4690 | *Haemophilus influenzae* |
| 1135 | *Leptotrichia buccalis* |
| 9143 | *Moraxella catarrhalis* |
| 2291 | *Mycoplasma pneumoniae* |
| 10036 | *Neisseria meningitidis* |
| 43665 | *Nocardia farcinica* |
| 16031 | *Pasturella multocida subsp. multocida* |
| 50090 | *Pseudomonas fluorescens* |
| 17058 | *Salmonella enterica subsp. enterica* |
| 20231 | *Staphylococcus aureus* |
| 2134 | *Streptococcus agalactiae* |
| 20575 | *Streptococcus constellatus subsp. consellatus* |
| 12492 | *Streptococcus infantis* |
| 6778 | *Streptococcus parasanguinis* |
| 20566 | *Streptococcus pneumoniae* |
| 18670 | *Streptococcus pseudopneumoniae* |
| 20565 | *Streptococcus pyogenes* |
| 20567 | *Streptococcus sanguinis* |
| 14222 | *Treponema denticola* |
| 44697 | *Mycobacterium psychotollerans* |
| 43990 | *Mycobacterium. bovis* |
| 19120 | *Actinomyces odontolyticus* |
| 20436 | *Bifidobacterium denticum* |
| 44287 | *Corynebacterium adiacens* |
| 9848 | *Granulicatella adiacens* |
| 8978 | *Haemophilus parainfluenzae* |
| 17633 | *Neisseria flavescens* |
| 19025 | *Prevotella buccae* |
| 20746 | *Rothia mucilaginosa* |
| 12643 | *Streptococcus mitis* |
| 20735 | *Veillonella dispar* |

### Data analysis.

**Sequence analysis in QIIME.**

Upstream analyses were performed using QIIME (Version 1.9.0)[^4^](#_ENREF_4). First the 8 base pair (bp) forward and reverse barcodes were combined into a single 16 bp unique identifier. Three prime ends of the sequences were quality trimmed and read through adaptors were removed using TrimGalore[^5^](#_ENREF_5). Forward and reverse reads were then combined into a single read using fastq-join[^6^](#_ENREF_6) with a requirement of a minimum overlap length of 200 bp with ≤ 10% misalignment. For any instances of mismatch, the base with the highest quality score was retained.

Next samples were de-multiplexed in order to assign sequences back to their original sample. Quality filtering was next performed with Phred scores assessed for each base and any read with an average score below the minimum quality threshold (< Q30) being removed. If 10 consecutive bases failed to meet the threshold, the read was truncated at this point. The resulting sequence was discarded if < 70% of consecutive bases in the combined read were ≥ Q30. During de-multiplexing, multiple sequencing runs containing samples from the same study group were combined into one dataset. Sequences generated from PhiX control library were removed after de-multiplexing by aligning surviving reads against the PhiX genome using the Burrows-Wheeler Alignment tool[^7^](#_ENREF_7).

Next, taking the resulting sequences, operational taxonomic units (OTUs) were assigned using an open reference approach with sequences clustered against the SILVA ribosomal RNA gene database (Version 115)[^8^](#_ENREF_8). OTU picking was performed in UCLUST (Version 1.2.22q) [^9^](#_ENREF_9) with a threshold of 97% sequence similarity, corresponding to a species level.

The most abundant sequence was picked as a representative for the OTU cluster. Sequences were aligned using the Python Nearest Alignment Space Termination (PyNAST) tool [^10^](#_ENREF_10) and a lanemask filtered alignment of the SILVA database [^8^](#_ENREF_8). Removal of chimeric sequences was performed using ChimeraSlayer [^11^](#_ENREF_11) and FastTree (Version 2.1.3) [^12^](#_ENREF_12) was used to construction of a phylogenetic tree. Species identity of the representative sequence was then assigned using UCLUST (Version 1.2.22q) [^9^](#_ENREF_9) and the SILVA database [^8^](#_ENREF_8). An OTU table was constructed detailing the OTUs present per sample. The OTU table, phylogenetic tree and representative sequences were uploaded into R for downstream analyses (performed in Phyloseq).

**Rarefaction versus random re-sampling with replacement**

The sequencing depth (the number of sequences per sample) differed widely between samples. In a longitudinal study the power comes from retaining as many sequential samples as possible and two different approaches were used in order to achieve this. Examining rarefaction curves, the majority of samples had reached an asymptote at 600 reads thus a rarefaction level of 600 reads was chosen. At this level, 38 samples (14%) had a sequencing depth below 600 reads and were removed.

Random re-sampling of a population with replacement is an alternative method of standardisation[^13^](#_ENREF_13). Using this method, species are randomly sampled from a population and diversity measures (e.g. richness and evenness) from the species sample are calculated. These species are then replaced back into the population sampled and the process repeated to a given number of iterations (e.g. 1,000 iterations). A matrix containing the mean, median, standard deviation and range of diversity scores is constructed for each sample, which can be used in statistical analyses. The advantage of this method is it does not require a minimum sequencing threshold, thus no samples are removed using this method, allowing more comparisons to be made between patients. A disadvantage is that rare species may fail to be sampled.

To ascertain whether random re-sampling with replacement (with 1,000 iterations) yields similar results to rarefaction (to 600 reads), alpha diversity results were compared between these two methods of standardisation. This showed that the results using random sampling with replacement were comparable with rarefaction. For example, a weak correlation was seen comparing changes in richness and age whether using rarefaction (t = 0.181, *P* < 0.001) or random re-sampling with replacement (t = 0.171, *P* < 0.001). Currently, however, this method cannot be used with the adonis function (PERMANOVA) in phyloseq, therefore random re-sampling with replacement was used with mixed effects modelling and rarefaction with adonis.

Alpha diversity was measured by species richness (the number of different species), evenness (the spread of species) and the Shannon Diversity Index (which gives a composite score of both richness and evenness). Non-linear mixed effects modelling using a negative binomial distribution were used to analyse changes in alpha diversity with age using the package glmmADMB in R.

Differences in beta-diversity was tested using the Bray-Curtis dissimilarity index[^16^](#_ENREF_16). The results are provided in a numerical distance matrix, which can then be used for further analyses. Multiple correlation testing to assess OTU level differences was performed using the Multtest package[^17^](#_ENREF_17).

The following clinical variables were tested for their influence on alpha and beta diversity: gender, genotype (homozygous versus heterozygous), Class mutation (I – III versus IV – VI), mode of delivery (vaginal delivery versus Caesarian section), type of feeds (breastfeeding versus formula feeding), pancreatic insufficiency, gastro-oesophageal reflux disease, upper respiratory tract symptoms (yes/no), lower respiratory tract symptoms (yes/no), antibiotics (oral, intravenous or nebulised), date of DNA extraction, sequencing plate number (for testing for batch effect).

### Results

**Figure S1: Frequency of throat swab (TS) sampling.**

**S1a: Illustration of the number of monthly samples collected.**

Each box in the grid represents a monthly sampling timepoint coloured by: red for baseline samples, yellow for follow-up samples, and blue for missing samples. Due to opportunistic sample collection, there was variability in sampling frequency. For some timepoints, multiple samples were collected.


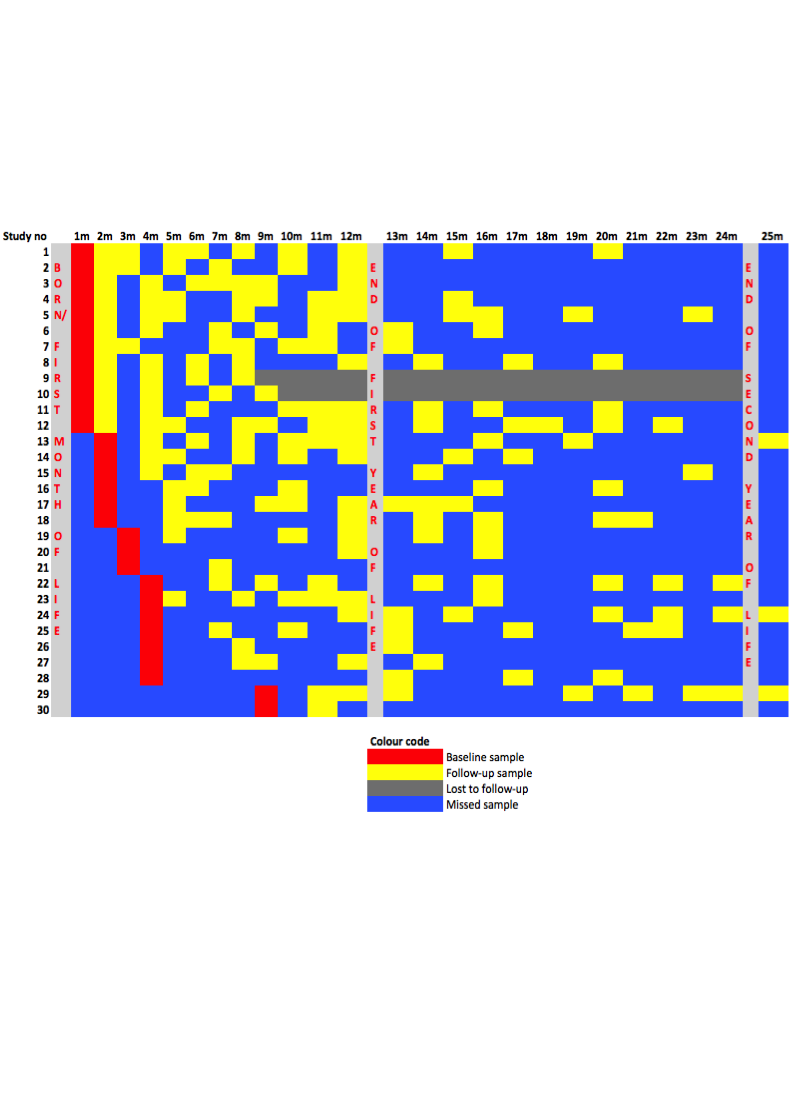


**S1b: Illustration of sample frequency clustered into age ranges.**

Due to greater frequency of sampling in the first year of life, samples were clustered into 2-month age ranges until 1 year of life and 3 month age ranges thereafter to allow the greatest number of comparisons between patients.


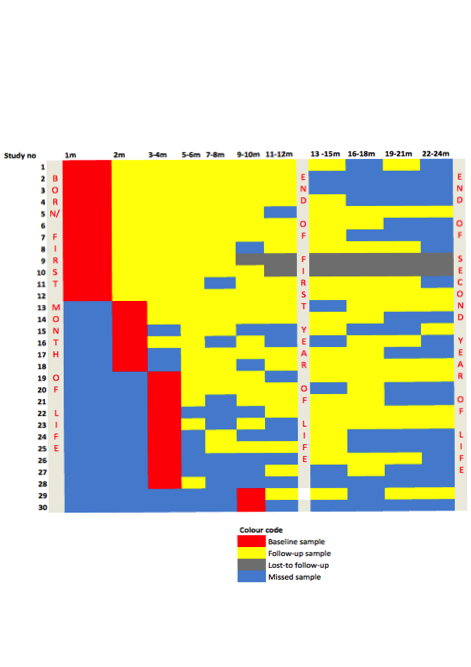


**Changes in the microbiota with the first pulmonary exacerbation or growth of *P. aeruginosa***

An exploratory analysis of the five patients having sequential samples revealed no significant difference in the relative abundance of any genera or OTU, bacterial load, or diversity between baseline, exacerbation, treatment and recovery samples (*P* > 0.05) (Figure S3).

Examining differences with the first growth of *P. aeruginosa*, there was a significant difference in beta diversity only between samples taken prior to, during or following *P. aeruginosa* growth, measured using Bray Curtis dissimilarity (r^2^ = 0.135, *P* = 0.036) and weighted UniFrac (r^2^ = 0.153, *P* = 0.025) but not the unweighted UniFrac score (r^2^ = 0.153, *P* = 0.068). There was no significant change in alpha diversity with growth and treatment of *P. aeruginosa*. There was no significant difference in relative abundance for any genus or OTU (*P_adj_* > 0.05) or total bacterial load (*P* > 0.05) (Figure S4).

**Figure S2: Changes in the relative abundance of the five most abundant genera with exacerbations.**

Changes in the relative abundance of *Streptococcus* spp., *Haemophilus* spp., *Granulicatella* spp., *Veillonella* spp. and *Prevotella* spp. at baseline (1 month prior to starting IV antibiotics), exacerbation (within 48 hours of starting IV antibiotics), treatment (at days 10-14 of IV antibiotics) and recovery (within 1 month of stopping IV antibiotics). All changes were non-significant (*P* > 0.05).

**Figure S3: Changes in the relative abundance (%) of five most common genera with the first growth of *Pseudomonas aeruginosa*.**

Changes in the relative abundance of *Streptococcus* spp., *Prevotella* spp., *Neisseria* spp., *Gemella* spp. and *Haemophilus* spp. in the month prior to *P. aeruginosa* growth (Pre-Pa), at the time of *P. aeruginosa* growth (Pa) and 1 month after treatment for *P. aeruginosa*, following successful eradication when bacterial cultures were negative (Post-Pa). All changes were non-significant (*P* > 0.05).

**References**
